# Modular Insulators: Genome Wide Search for Composite CTCF/Thyroid Hormone Receptor Binding Sites

**DOI:** 10.1371/journal.pone.0010119

**Published:** 2010-04-09

**Authors:** Oliver Weth, Christine Weth, Marek Bartkuhn, Joerg Leers, Florian Uhle, Rainer Renkawitz

**Affiliations:** Institute for Genetics, Justus-Liebig-University Giessen, Giessen, Germany; Institute of Genetics and Molecular and Cellular Biology, France

## Abstract

The conserved 11 zinc-finger protein CTCF is involved in several transcriptional mechanisms, including insulation and enhancer blocking. We had previously identified two composite elements consisting of a CTCF and a TR binding site at the chicken *lysozyme* and the human *c-myc* genes. Using these it has been demonstrated that thyroid hormone mediates the relief of enhancer blocking even though CTCF remains bound to its binding site. Here we wished to determine whether CTCF and TR combined sites are representative of a general feature of the genome, and whether such sites are functional in regulating enhancer blocking. Genome wide analysis revealed that about 18% of the CTCF regions harbored at least one of the four different palindromic or repeated sequence arrangements typical for the binding of TR homodimers or TR/RXR heterodimers. Functional analysis of 10 different composite elements of thyroid hormone responsive genes was performed using episomal constructs. The episomal system allowed recapitulating CTCF mediated enhancer blocking function to be dependent on poly (ADP)-ribose modification and to mediate histone deacetylation. Furthermore, thyroid hormone sensitive enhancer blocking could be shown for one of these new composite elements. Remarkably, not only did the regulation of enhancer blocking require functional TR binding, but also the basal enhancer blocking activity of CTCF was dependent on the binding of the unliganded TR. Thus, a number of composite CTCF/TR binding sites may represent a subset of other modular CTCF composite sites, such as groups of multiple CTCF sites or of CTCF/Oct4, CTCF/Kaiso or CTCF/Yy1 combinations.

## Introduction

The conserved 11 zinc-finger protein CTCF is involved in several transcriptional mechanisms, such as gene activation [1], gene repression [2], [3] and enhancer blocking [4]–[13]. In vertebrates, CTCF is the only identified protein that is able to bind to insulators and to mediate enhancer blocking [4]. Insulators block the action of enhancers when positioned between enhancer and promoter, thereby preventing inappropriate action of enhancers on neighbouring genes. The CTCF-mediated enhancer blocking can be constitutive, for example in the locus control region of the *β-globin* genes [4]. In other cases, regulation of enhancer blocking has been described at the level of DNA-binding. It has been shown that CTCF is not able to bind to those binding-sites that include methylated CpGs. A well characterized system for such a regulation is the imprinting control region (ICR) of the *Igf2/H19* locus. At this lCR the binding-site for CTCF on the paternal allele is methylated. This leads to inhibition of DNA-binding of CTCF and thereby to the relief of enhancer blocking [5]–[7], [9], [14]–[17]. Another mechanism in the regulation of DNA binding of CTCF is in the context of transcription, when RNA polymerase transcribes through the CTCF binding site. This activity dislocates CTCF from the DNA [18]. Previously, we had identified a different type of regulation of enhancer blocking. This involves thyroid hormone (T3), which can regulate enhancer blocking by CTCF [8]. A subset of CTCF binding sites is found next to thyroid hormone response elements, such as in the composite elements located in the rat element 144, the mouse *c-myc* gene and the human *APP* gene. The thyroid hormone receptor (TR) is a member of the nuclear hormone receptor family of transcription factors. TR associates with proteins that possess histone acetyltransferase activity (HAT) in the presence of its ligand T3. In the absence of T3, TR is complexed with enzymes that mediate histone deacetylation. For the composite element consisting of a CTCF and a TR binding site within the chicken *lysozyme* upstream silencer, CTCF binds to footprint 1 (F1), and TR binds as a homodimer or heterodimer with the retinoid-X-receptor (RXR) to footprint 2 (F2). For this element it has been demonstrated that T3 mediates relief of enhancer blocking and that activation occurs even though CTCF remains bound to its binding site [8]. Furthermore, ChIP analysis of the *lysozyme* upstream region revealed that histone H4 is acetylated at the CTCF binding site. Loss of enhancer blocking by the addition of T3 led to increased histone acetylation, not only at the CTCF site, but also at the enhancer and the promoter [8]. Nuclear hormone receptor binding sites next to important CTCF sites at the *Igf2/H19* locus have been found, but no functional effects with respect to insulation, enhancer blocking or imprinting could be shown [19].

Here we wanted to determine whether CTCF and TR combined sites are representative of a general feature of the genome, and whether such sites are functional in regulating enhancer blocking.

## Results

### Genome wide search for binding sites for TR and for CTCF

**Figure 1:**
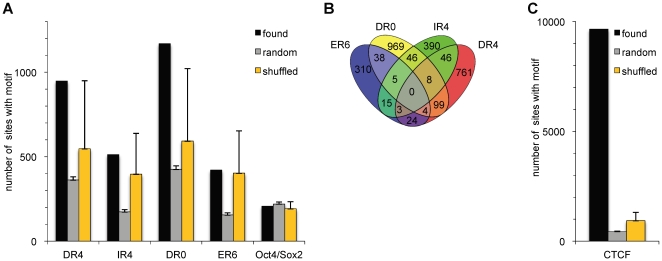
Frequency of TREs at CTCF binding regions. 13720 CTCF binding regions [20] from −500 to +500 bp relative to the respective peak centers were scanned with the PSSMs (DR4, DR0, IR4, ER6 and Oct4/Sox2) downloaded from TRANSFAC and Li et al. [55]. (A) Depicted is the number of 1000 bp regions that were marked by one or more of the respective motifs (found). Those results were compared to 3500 sets of 13720 random 1000 bp sequences from the repeat masked human genome (random). Shown is the mean number of sites marked by the respective motif. To control for potential sequence bias in the CTCF bound regions we shuffled the matrices keeping the information content constant. Shuffled matrices were used to scan the 13720 CTCF binding sequences (yellow). Error bars indicate the maximum deviation from the mean. (B) Venn diagram presentation of all TREs identified in CTCF regions, indicating the frequency of single, double and triple TRE occurrences at CTCF regions. (C) The positive control for this procedure shows the highly significant detection of the CTCF consensus (found) in contrast to random sequences (random) as in (A).

In order to analyze whether the occurrence of composite CTCF and TR binding sites is a common feature for many or most CTCF binding sites, or whether this is limited to a subset of CTCF sites, we examined the distribution of CTCF and thyroid hormone receptor binding elements in a genome-wide fashion. CTCF binding has been mapped in a number of experimental approaches. A consensus binding sequence was derived from these studies, which was found in a large number of the identified in vivo binding regions [20]. In contrast to CTCF, no genome-wide binding data are available for the thyroid hormone receptor. Nevertheless it is known that the thyroid hormone receptor binds to DNA through conserved dimers of the hexanucleotide sequence AGGTCA (thyroid hormone response elements: TRE). The latter can be found in different arrangements, such as direct repeats with 4 or 0 nucleotides spacing (DR4 or DR0), inverted repeats spaced by 4 nucleotides (IR4) and everted repeats with a 6 nucleotide spacing (ER6) [21]. We constructed position specific scoring matrices (PSSM) based on half-site models derived from the TRANSFAC-database and used the Patser tool [22] to identify potential TR binding sites throughout the repeat-masked human genome. Performance of our PSSM-based approach was tested against two well-known motif prediction tools that are especially well suited for the detection of occurrences of nuclear hormone receptor binding sites: NHR-scan and NUBIscan [23], [24]. In order to compare the different detection methods we ran comparisons on randomly selected 1 Mb genomic regions with the following setting: raw score >0.7 and p<0.05 (NUBIscan), score >6.6, p<10^−13^ (Patser), default parameter (NHR-scan). Despite of the different scoring algorithms we detected identical motifs in >80% of the cases. Furthermore we were able to detect a number of previously identified TREs such as the DR4 element in the myc N site ([8] data not shown). To test whether the candidate motifs might be enriched in CTCF binding regions as compared to random genomic sequences, we scanned 13720 CTCF bound regions of 1000 bp length ([20]; +/− 500 bp from annotated binding region center) and compared them to 3500 times 13720 random genomic control regions. As shown in Figure 1A we could detect various half-site arrangements in a subset of CTCF binding regions, ranging from 2.9% (400 ER6 sites) to 8.7% (1200 DR0 sites) of the total number of CTCF binding regions. We included the binding sequence for Oct4/Sox2 as a control, which has been shown to occur in functionally relevant combinations with CTCF [25], but for which no significant correlation of CTCF and Oct4/Sox2 binding was observed [26]. Here we also find that the number of CTCF regions with Oct4/Sox2 binding sequences is similar to random sequences (Fig. 1A). In contrast, the occurrence of TRE typical half site arrangements (DR4, DR0, ER6 and IR4; Fig. 1A) was slightly increased as compared to random sequences. In order to control whether this observation indeed represents an enrichment of NR motifs next to CTCF binding sites or a bias in the underlying base composition, we applied an additional control experiment: we shuffled the base positions of the individual TRE motifs and used the scrambled PSSMs to scan the 13720 CTCF binding sites. In contrast to our previous experiment, we did not find a significantly better performance of the real PSSM over scrambled ones (p-values: 0.17; 0.3; 0.44; 0.44 for DR4, IR4, DR0 and ER6 respectively). Therefore we conclude that there is no significant enrichment of TREs next to CTCF binding sites throughout the genome. This does not rule out the principal presence of functional combinations of CTCF/TR binding elements.

Often, several TREs occurred simultaneously in CTCF regions, with up to three different TRE types in one CTCF region as depicted in a Venn diagram (Fig. 1B). A total of 2430 CTCF regions were detected that harbored at least one of the four different TRE sequences. As a positive control for our analysis we tested the number of cases where the CTCF consensus was found in the CTCF binding regions (Fig. 1C). In about 70% of the CTCF binding regions a consensus was detected, which is in agreement with published data [20]. As expected, we found 10-fold fewer instances in random sequences or using the scrambled versions of CTCF PSSM (Fig. 1C).

Taken together these data indicate that TR binding motifs mark a fraction of CTCF binding regions. The frequency of composite CTCF/TR elements is not higher than that detected with scrambled PSSMs. This finding does not support a general role for the TR. Despite of this, individual composite elements could be functional as exemplified with the CTCF/Oct4/Sox2 cases [25], [26]. From these data we reasoned that although individual examples for composite CTCT/TR binding elements are not a general feature of CTCF binding regions, a number of functional TRE motifs in the vicinity of CTCF binding sites might exist.

**Figure 2:**
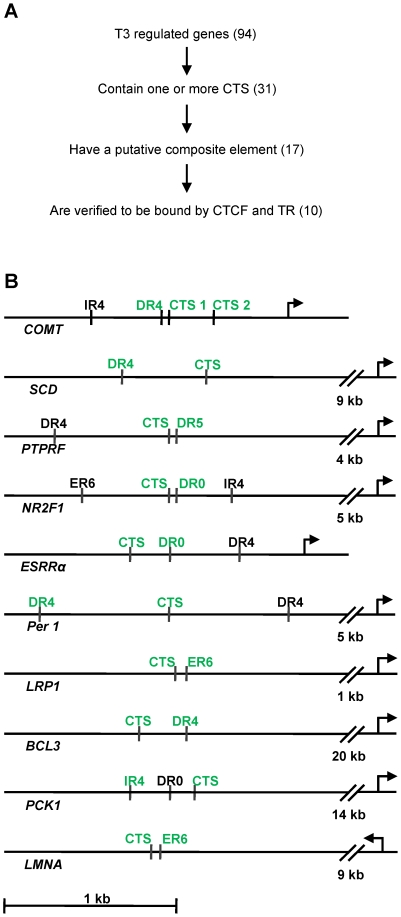
Schematic representation of genes with a composite element. (A) 94 T3 responsive genes conserved in mice and man searched for CTCF composite elements (see main text). (B) Each diagram presents a region of the indicated gene. Putative binding sites for CTCF (CTS), binding sites for TR (IR4, DR4, DR5, ER6 or DR0) and the transcriptional start site (arrow) are shown. Elements being shown to bind TR or CTCF (see Fig. 3) are indicated in green.

In order to functionally verify the predicted composite CTCF/TR binding sites, we concentrated on those genes which have been shown to be regulated by T3 in the mouse or in the rat [27]–[30] (Fig. 2A). From these we screened 94 genes conserved in human for the presence of a CTCF binding site (CTS) within or outside of the transcribed region including 40 kb of flanking sequence upstream and downstream. For CTCF we again used the dataset of genome wide CTCF binding [20]. We identified 31 T3 responsive genes with one or more CTCF binding sites, whereas 63 did not contain a known CTCF target site. In order to predict TR binding sites within the T3 responsive genes we used the NubiScan algorithm, which allows the prediction of nuclear receptor response elements [23]. We focused on the direct repeats DR0 and DR4, as well as on the palindromes IR4 and ER6, which are known to bind either TR homo or TR/RXR heterodimers [21]. We divided each of the gene loci into the 5′region and the intronic plus 3′region. Of the 31 gene loci with a CTCF site, 23 harbor CTCF sites in the 5′region. For these a TR binding site in a range of +/− 1 kb flanking a CTS could be identified in 13 cases. Within the intronic and 3′ CTS group, TREs could be identified in the vicinity of a CTS in 4 cases. Thus, a total of 17 gene loci harbor a composite TRE/CTS. Based on the score of binding site predictions, we chose 10 (Fig. 2) for experimental analysis.

### Composite elements are bound by TR and CTCF

**Figure 3:**
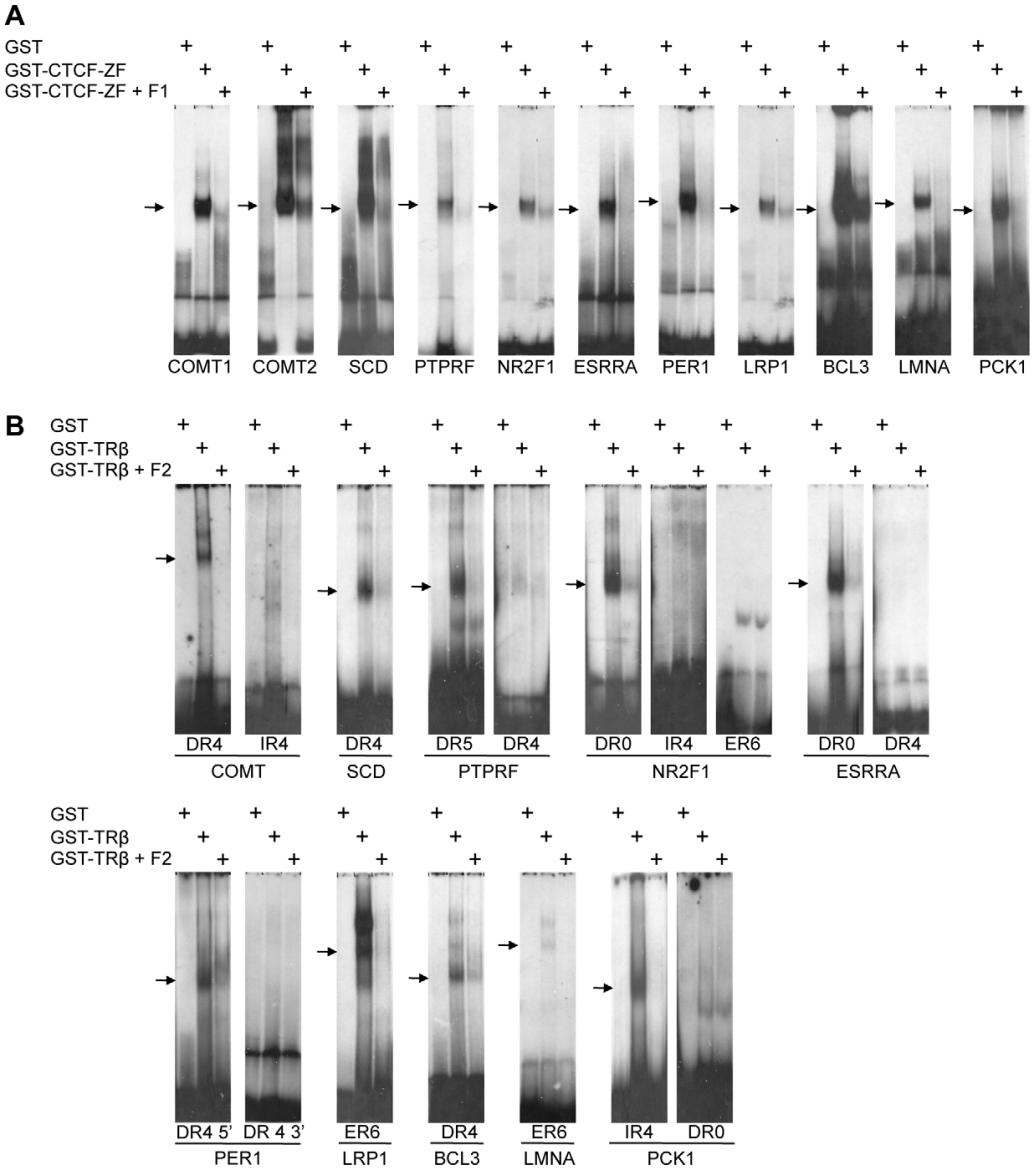
In vitro binding assays (EMSA) show direct binding of CTCF and TRβ to predicted target sites. EMSA experiments were performed using E.coli expressed GST, GST-CTCF-ZF (A) or GST-TR (B) with the indicated radioactively labeled probes. For competition experiments a 50-fold molar excess of non-labeled probes were used. These were either an unrelated CTCF binding site (F1) or an unrelated TR binding site (F2). Arrows mark the CTCF or TR specific shift.

In vivo binding of CTCF to the gene regions shown in Figure 2 has been documented by genome wide binding analysis [20]. The in vivo response to T3 for the selected genes has been shown as well [27]–[30]. In order to identify the precise binding sites and to validate the TRE predictions, we tested TR and CTCF binding in vitro using the electrophoretic mobility shift assay (EMSA). For CTCF analysis we used a fusion of GST with the zinc finger domain of CTCF (GST-CTCF-ZF) expressed in E.coli. The DNA probes were selected by CTCF binding site prediction for each of the 10 genes chosen (Fig. 2B). In all cases, GST-CTCF-ZF resulted in a retarded band (Fig. 3A). For the *Catechol-o-methyl-transferase* gene (*COMT*) two CTS are predicted at the downstream promoter, both of which are bound as well. The bound complex could be competed by an unrelated CTCF binding site (F1), whereas the negative control, GST protein expressed in E.coli, resulted in no specifically retarded complex.

In order to confirm that these CTS elements are indeed the same sequences identified by genome wide CTCF binding analyses [20], [31], we precipitated HeLa cell chromatin with an antibody directed against CTCF. Exemplarily, we analyzed the precipitate for the presence of five of the sequences shifted by CTCF and found all to be bound in vivo as well (Fig. S1).

Similarly, we analyzed binding of a GST-TR fusion to the predicted binding sites of these T3 responsive genes. Binding site prediction in several cases identified two or more potential TRE elements. These have half site arrangements of the type DR0, DR4, IR4, ER6 or an atypical [32] DR5 site (Fig. 2B). The DR4 element shows a clear shift in mobility when incubated with GST-TR at the *COMT* downstream promoter, but is not shifted with GST alone (Fig. 3B). The binding is specific as competition by a known TR binding site (F2) abolished protein binding, and competition with a mutated F2 site did not (Fig. S2). No shift is visible when the IR4 element of the *COMT* gene is incubated with GST-TR, indicating that this sequence is not bound by TR in vitro (Fig. 3B).

We examined all predicted TR binding sites and could identify several new TR target sites. A detailed summary of these results is shown in Table 1 and the confirmed binding sites are indicated by green lettering (Fig. 2B). In general, all of these 10 identified regions at T3 responsive genes harbor a functional TR binding element, validating the PSSM procedure to predict TR binding sites as described above. Chromatin precipitation for TR could not be performed because of the lack of suitable antibodies. The gene products are involved in neurotransmission, physiology, signalling, transcriptional regulation and cell cycle. Four genes are up-regulated and six are down-regulated by T3. CTCF knockdown [33] affects three of these genes, with two of them being repressed and one being induced (Table 1).

**Table 1:** Ten T3 regulated genes harbor CTCF/TR composite elements.

Name	Characteristics	Function	Regulation	Ref.
*COMT*	Catechol-O-methyltransferase is found in two forms, a soluble form (S-COMT) and a membrane-bound form (MB-COMT).	COMT catalyzes the transfer of a methyl group from S-adenosylmethionine to catecholamines, including the neurotransmitters dopamine, epinephrine, and norepinephrine.	Down regulated upon T3 treatment.	[29]
*SCD*	Four Stearoyl-CoA desaturase isoforms, Scd1 through Scd4, have been identified in mouse. 2 SCD isoforms, SCD1 and SCD5, have been identified in human.	SCD is an iron-containing enzyme that catalyzes a rate-limiting step in the synthesis of unsaturated fatty acids.	Up regulated upon T3 treatment.	[29]
*PTPRF*	Protein tyrosine phosphatase receptor type F possesses an extracellular region, a transmembrane region, and two tandem intracytoplasmic catalytic domains.	PTPs are signaling molecules that regulate a variety of cellular processes including cell growth, differentiation, mitotic cycle, and oncogenic transformation.	Up regulated upon T3 treatment.	[27]
*NR2F1*	Nuclear receptor subfamily 2, group F (COUP-TF) is a nuclear receptor and binds to both direct repeats and palindromes of the 5′-AGGTCA-3′ motif.	NR2F1 transcription factor binds to the ovalbumin promoter and, in conjunction with another protein (S300-II) stimulates initiation of transcription.	Down regulated upon T3 treatment. Slightly induced after CTCF knockdown.	[27], [33]
*ESRRA*	Estrogen-related receptor alpha is a nuclear receptor that is closely related to the estrogen receptor.	ESRRα function has been demonstrated in the regulation of a variety of genes including lactoferrin, osteopontin, medium-chain acyl coenzyme A dehydrogenase (MCAD) and thyroid hormone receptor genes.	Down regulated upon T3 treatment.	[27]
*PER1*	Period1 is expressed in a circadian pattern in the suprachiasmatic nucleus, the primary circadian pacemaker in the mammalian brain.	PER1 Influences clock function by interacting with other circadian regulatory proteins and transporting them to the nucleus.	Down regulated upon T3 treatment.	[29]
*LRP1*	Low density lipoprotein-related protein 1 is required for early embryonic development and involved in cellular lipid homeostasis.	LRP1 is an endocytic receptor involved in endocytosis and in phagocytosis of apoptotic cells.	Down regulated upon T3 treatment.	[28]
*BCL3*	B-cell lymphoma 3 is a proto-oncogene candidate translocated into the immunoglobulin alpha-locus in some cases of B-cell leukemia.	BCL3 could be a transcriptional activating factor. Inhibits translocation of NF-kappa-B p50 subunit to the nucleus.	Up regulated upon T3 treatment. Repressed after CTCF knockdown.	[28], [33]
*PCK1*	Phosphoenolpyruvate carboxykinase 1 is a main control point for the regulation of gluconeogenesis.	PCK1, along with GTP, catalyzes the formation of phosphoenolpyruvate from oxaloacetate, with the release of carbon dioxide and GDP.	Up regulated upon T3 treatment.	[30]
*LMNA*	Lamin A is a family member of matrix proteins that are highly conserved in evolution.	During mitosis, the lamina matrix is reversibly disassembled as the lamin proteins are phosphorylated. Lamin proteins are thought to be involved in nuclear stability, chromatin structure and gene expression.	Down regulated upon T3 treatment. Slightly repressed upon CTCF knockdown.	[28], [33]

### Hormone sensitive enhancer blocking on episomes

**Figure 4:**
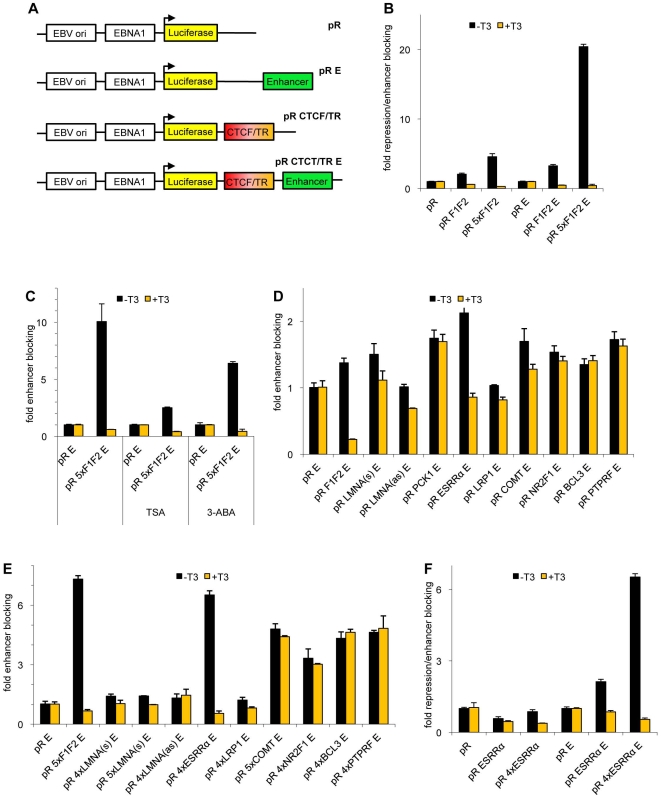
F1F2 and ESRRα composite elements mediate hormone sensitive enhancer blocking on episomes. (A) Schematic representation of the insulator-enhancer-reporter arrangement of the episomal vectors. The composite insulator sequence (CTCF/TR) is usually integrated as a single insert, or as 4x or 5x multimers as indicated for the constructs used. Cells transfected with the indicated episomes were incubated for 48 h in the absence or presence of T3. (B) Efficient enhancer blocking depends on the presence of the enhancer. The repressive effects cannot be attributed to direct promoter repression. Transfection was in N2aβ cells. (C) TSA and 3-ABA were added 8 h or 28 h after transfection, respectively. Transfection was in N2aβ cells. (D–F) HepG2 cells were additionally cotransfected with 0.2 µg TRβ expression vector. (F) Comparison of the enhancer-less constructs with the enhancer constructs demonstrate that the enhancer blocking activity is dependent on the enhancer.

In order to functionally analyze hormone regulated enhancer blocking, we used a test system based on episomal vectors. The episomal system has the advantage that the reporter DNA is not integrated into the genome, and furthermore that the DNA is packaged into chromatin [34]. Therefore, this system allows the study of transcriptional regulation on properly formed chromatin independent of the integration locus in the genome. Using stably integrated DNA for the CTCF/TR composite element of the chicken *lysozyme* gene (F1F2 element), we have previously shown that CTCF mediated enhancer blocking is T3 sensitive [8]. We wondered whether we could use the F1F2 element as a crucial test system to study composite CTCF/TR elements with episomes. Enhancer function and enhancer blocking is quantified by measuring the activity of the *luciferase* reporter gene. Transfection of a series of episomes into N2aβ cells, which express TRβ, revealed that F1F2 mediates enhancer blocking, which is dependent on both the presence of the F1F2 element and enhancer (Fig. 4A, B). A single F1F2 element mediates weak enhancer blocking, whereas a five times multimerized element (5xF1F2) mediates a more than five-fold robust enhancer blocking. The backbone vectors pR or pR E do not change the luciferase activity after the addition of thyroid hormone, whereas in contrast the pR F1F2 E or pR 5xF1F2 E constructs fully abolish enhancer blocking. Again, this effect depends on the presence of the enhancer, since the enhancer-less constructs show only a weak T3 response.

It is known that the CTCF-mediated repression function is partially sensitive to the HDAC-inhibitor Trichostatin A (TSA) [35]. Furthermore, it has been shown that CTCF is able to recruit HDAC activity from HeLa extract and interacts in vitro with Sin3A [35]. We analyzed whether TSA has an influence not only on repression, but also on the enhancer blocking function of CTCF. Incubation of the cells transfected with the enhancer blocking F1F2 episome with TSA resulted in a reduction of enhancer blocking activity to about 25% as compared to untreated cells (Fig. 4C). This indicates that HDACs are not only involved in the repression function of CTCF, but also in enhancer blocking.

Another modification of CTCF, poly (ADP)-ribose (PAR) binding, has been shown to be essential for mediating enhancer blocking and insulation [36], [37]. If indeed the episomal enhancer blocking assay is able to fully simulate enhancer blocking of endogenous genomic insulators, PAR modification should affect the episomal assay. Therefore we used the inhibitor of PAR polymerases, 3-Aminobenzamide (3-ABA), for the transfection experiments. The CTCF-mediated enhancer blocking is decreased to about 70% upon the addition of 3-ABA (Fig. 4C).

Thus, the episomal system used in these experiments fully mimics the enhancer blocking function of insulators integrated in the genome.

### T3 responsive and non-responsive composite CTS elements

Since the episomal system proved to be suitable for the analysis of composite CTCF/TR binding sites, we generated enhancer blocking episomes with the newly identified composite elements. HepG2 cells were chosen as a test system because most T3 responsive genes found are expressed in liver. Single insertions of LMNA, PCK1, ESSRA, COMT, NR2F1, BCL3 and PTPRF mediated weak enhancer blocking (Fig. 4D). The arrangement of the LMNA composite element relative to the promoter is, in contrast to the other genes, downstream of the promoter. Therefore, we also tested the antisense orientation in the episome (pR LMNA(as) E), which did not show any enhancer blocking activity. Two of the elements, the positive control F1F2 and ESRRA, mediated enhancer blocking in the absence of T3, whereas T3 fully abolished this activity. In order to detect possible weak effects in enhancer blocking and T3 mediated relief from enhancer blocking, we generated four or five times multimerized composite CTCF/TR binding sites. In six cases, F1F2, ESRRA, COMT, NR2F1, BCL3 and PTPRF a significant increase in enhancer blocking activity could be seen (Fig. 4E). In contrast to these new enhancer blocking elements, the composite CTS of LRP1 and LMNA in either orientation did not confer enhancer blocking. In all cases we challenged the luciferase activity by incubation with T3. The composite elements of PCK1, COMT, NR2F1, BCL3 and PTPRF mediated constitutive enhancer blocking, which could not be relieved by T3 (Fig. 4D, E). In contrast, the ESRRA element as a single insertion or when multimerized four times resulted in a T3 mediated complete release of enhancer blocking (Fig. 4D, E). We could not detect any repressive/silencing effects of the ESRRA element on the luciferase promoter using the enhancer-less episomal vectors (Fig. 4F). Thus, the effects seen are dependent on an enhancer and can therefore be defined as enhancer blocking.

Taken together the ESRRA element is a new composite element mediating hormone sensitive enhancer blocking and together with the F1F2 element is suitable for further studies.

### Functional CTS composite elements depend on both, TR and CTCF

**Figure 5:**
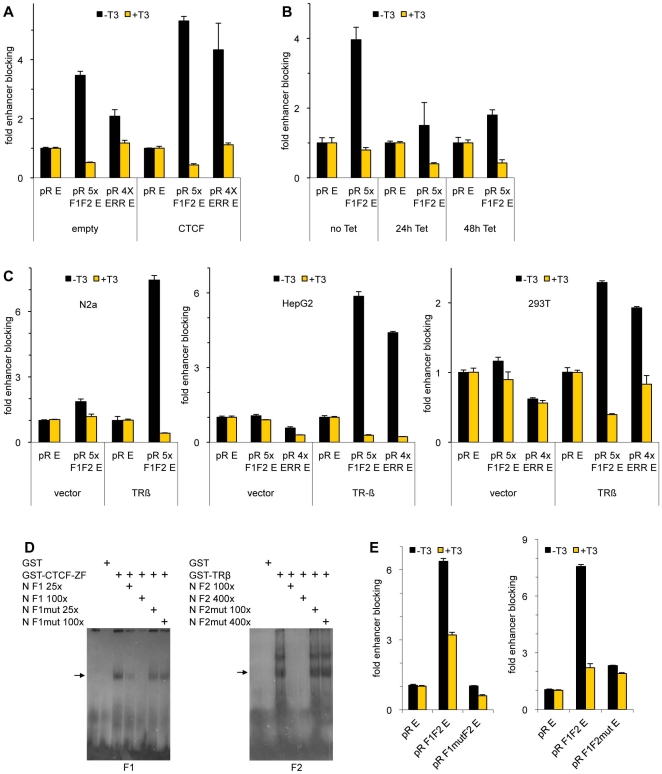
CTCF and TR synergize in enhancer blocking. Cells transfected with episomes were incubated for 48 h in the absence or presence of T3. ESRRA  =  abbr. ERR. (A) 293T cells were transfected with the indicated episomes, a TRβ expression vector, and with either the CTCF expression vector (CTCF) or an empty vector (vector). (B) A 293T cell clone containing a stably integrated vector with Tet-inducible expression of shRNA against CTCF was transfected with the indicated reporter episome and the TRβ expression vector. The cells were incubated with tetracycline for 0, 24 or 48 h. (C) Indicated cell lines expressing either a low amount of or no TRβ were transfected with reporter episomes and either empty vector (vector) or TRβ-expression (TRβ). (D) EMSA experiments were performed using E.coli expressed GST, GST-CTCF-ZF and GST-TR and radioactively labeled F1/F2 probe. For competition experiments, non-labeled probes (N) were used in amounts as indicated. (E) Either the mutated F1 (F1mutF2) or the mutated F2 (F1F2mut) were tested in N2a cells in the presence of TR-expression vector.

Since T3 relieves the enhancer blocking activity from the F1F2 and the ESRRA composite elements, we wanted to address a possible contribution of the TR to enhancer blocking in the absence of T3. As a control we tested the contribution of CTCF to enhancer blocking by utilizing 293T cells, which allow for high level expression. Since CTCF is expressed in all cell types, we used transfection conditions resulting in a low level of enhancer blocking. Co-transfection of an expression vector for CTCF leads to a significant increase in enhancer blocking on the F1F2 and ESRRA elements, indicating that CTCF is at least contributing to enhancer blocking (Fig. 5A). In a complementary approach we reduced the amount of CTCF using a tetracycline inducible shRNA-mediated knock-down of CTCF. After 48 hours this antisense approach resulted in a down regulation of CTCF protein to about 50% (Fig. S3). This decreased expression of CTCF led to a 50% decrease in enhancer blocking (Fig. 5B), again confirming that CTCF is involved in the observed enhancer blocking. The addition of T3 completely abolishes enhancer blocking both when the blocking is increased after CTCF overexpression, and is decreased after CTCF knockdown.

We then tested the influence of TR on enhancer blocking. We transfected F1F2-containing episomes into N2a cells, that is a cell line which, in contrast to N2aβ cells, does not express TR. This allows for the testing of TR mediated effects upon TR expression. We observed that CTCF alone is not sufficient to mediate enhancer blocking, whereas the cotransfection of a TR expression vector restores enhancer blocking (Fig. 5C). The same is true for the ESRRA element containing episomes when transfected into HepG2 cells. These cells have a reduced amount of TR resulting in a reduced T3 induction of transfected DR4 luc reporter DNA. Full DR4 reporter activity can only be achieved upon overexpression of TRβ (data not shown). Therefore, we cotransfected the TRβ expression vector and the episomal reporter construct. This resulted in about a 5-fold enhancer blocking using the ESRRA or the F1F2 elements (Fig. 5C). Likewise, the enhancer blocking activity of both elements, F1F2 and ESRRA, was dependent on the expression of TRβ when using 293T recipient cells.

This strong impact of the unliganded TR on enhancer blocking was unexpected. Therefore we wanted to understand the individual contribution of CTCF and TR to enhancer blocking and mutated the individual binding sites within the F1F2 element. To abolish CTCF binding to its cognate binding site, 2 nucleotides within the F1 binding site of CTCF were changed (AA – GG) and tested in band shift assays using *E.coli* expressed CTCF-zinc finger domain fused to GST (GST-CTCF ZF). The F1wt binding site is shifted by GST-CTCF ZF. This shift can be competed with an excess of unlabelled F1wt site but not by the same excess of unlabelled mutated F1 site (Fig. 5D). To test the functional consequence on enhancer blocking by the mutated binding site, we performed transfection experiments with episomes containing the F1F2 or the F1mutF2 binding sites. These experiments revealed that in contrast to the wt F1F2 element, the F1mutF2 binding site is not capable of mediating significant enhancer blocking (Fig. 5E).

To test the effects of TR on CTCF mediated enhancer blocking, we mutated the two half sites of the F2 element, which is characterized by an everted palindrome spaced by 6 nucleotides. We performed band shift assays with the wt F2 sequence as a probe, and bound GST-TR could be fully competed by the addition of unlabelled wild type probe. In contrast, the mutated unlabelled F2 element could not compete this shift even at a high molar excess (Fig. 5D). To test the functional consequences of the mutated F2 in enhancer blocking we introduced this mutation into the F1F2 enhancer blocking construct. Transfection experiments with constructs containing F1F2wt or F1F2mut showed that mutation of the F2 binding site did not only abrogate any T3 effect, but also impaired enhancer blocking (Fig. 5E).

These results demonstrate that enhancer blocking depends on the presence of two factors, CTCF and TR. CTCF alone is not able to mediate enhancer blocking on these composite elements, an unliganded TR synergizes with CTCF and this synergism can then be abolished by the presence of T3.

**Figure 6:**
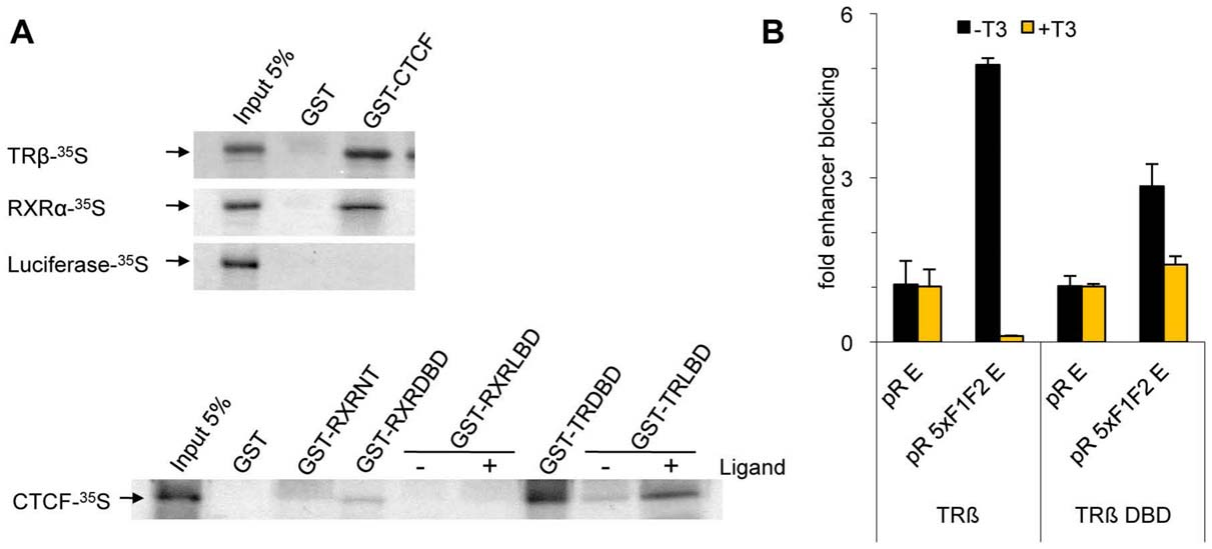
TR requires more than just the CTCF interaction domain to mediate enhancer blocking. (A) GST-pulldown experiments were carried out using the indicated *E.coli* expressed GST-fusion proteins. (B) N2a cells were transfected with the indicated episomes and expression vector for full length TRβ or for TRβ DBD and incubated in the absence or presence of T3.

TR interaction with CTCF has been shown [38]. The physiological TR/DNA complex may be a TR homodimer or a TR and RXR heterodimer [21]. Therefore we tested whether CTCF interacts with TR, RXR, or with both. We performed GST pull-down assays with GST fused CTCF and ^35^S-radiolabeled TR or RXR. The pulldown experiments clearly showed that both TR and RXR interact with CTCF (Fig. 6A). To identify domains of the receptor required for interaction we generated GST fusions with the individual DNA binding domains (DBD) of TR and RXR or with the ligand binding domain (LBD). The TR-DBD binds very efficiently and seems to harbor the main interaction interface. The N-terminal domain of RXR binds to CTCF to a lesser extent,the ligand binding domain shows no CTCF binding in the case of RXR, and only weak binding in the case of TR (Fig. 6A).

Thus the DBD of TR harbors two potentially important functions for enhancer blocking, such as DNA binding and an interface for the physical interaction with CTCF. Therefore we functionally tested the TR-DBD for enhancer blocking. We used cells that do not express endogenous TR (N2a cells) and expressed either full length TR or the DNA binding domain of TR. In contrast to the TR-DBD, only full length TR results in strong enhancer blocking of the F1F2 episome (Fig. 6B). This indicates that the TR-DBD is not sufficient for enhancer blocking and that other domains outside the DNA binding domain of TR are needed as well to mediate enhancer blocking of the CTCF bound module and the unliganded TR.

## Discussion

CTCF binding sites have been found next to thyroid or steroid receptor binding sites in several cases [8], [39], [40]. For a subset of these sites we could demonstrate that enhancer blocking is regulated by thyroid hormone [8]. In other cases, clustered binding sites for steroid receptors next to highly conserved CTCF sites in the *Igf2/H19* locus, a function could not be demonstrated [19]. Therefore, we wanted to know the frequency of combined binding sites for CTCF and the thyroid hormone receptor in the genome.

Of 13720 CTCF bound regions we found about 18% to harbor a potential TRE. This ratio is slightly higher than random sequences associated with CTCF bound regions. Clearly, the positive control to search for the CTCF consensus within these regions revealed a significant 22 fold higher frequency as compared to random controls. We checked the PSSMs for different half-site models against whole genome datasets of the estrogen receptor (ER) and the retinoic acid receptor alpha (RARα) [40], [41] in order to validate our approach in identifying such elements next to CTCF sites. In these calculations we found that the respective half-site models (IR3 and DR5) showed no significant enrichment when we compared the genome-wide binding datasets with control sequences (data not shown).

Previous analysis of estrogen receptor binding sites in comparison with CTCF binding revealed that CTCF site distribution strongly correlated with gene density in contrast to ER sites [20]. Based on the CTCF distribution, the genome can be dissected into sequence blocks framed by CTCF sites. About 3000 such CTCF blocks have been found to contain ER binding sites [42]. The authors concluded that CTCF confines the distal action of the estrogen receptor. This would argue for a clear separation of CTCF and ER sites in function and space for most of the binding sites. This is in line with the finding that of 1600 ER sites analyzed, about 100 colocalized with the CTCF motif [40]. Overall, the ratio of composite CTCF and TR binding sites relative to the total number of TR or CTCF sites is within the same magnitude as found for ER and CTCF.

**Figure 7:**
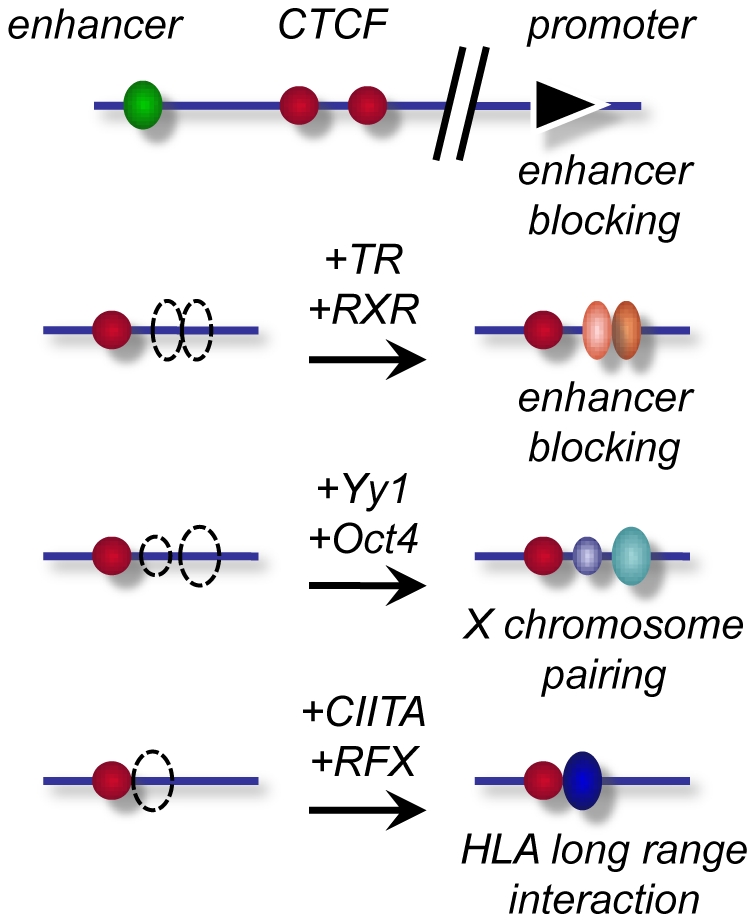
Modular CTCF binding sites. Enhancer blocking elements may require multiple CTCF sites for optimal function, as exemplified at the *H19* locus or the *Xist* and *Tsix* locus [15], [50], [51] (top). The composite elements of the *lysozyme* and *ESRRA* genes require binding of RXR and/or TR for enhancer blocking (second row). Long range chromatin interaction is not only required for enhancer blocking, but for X chromosomal pairing and HLA locus interaction as well. In addition to CTCF, this involves Yy1 and Oct4 [25], [53], [62] (third row) or CIITA at the CTCF site and RFX at the interacting site [63], [64] (bottom row).

Despite the low frequency of composite CTCF/TR binding elements, these can be important and functional. The importance of low frequency composite elements has been illustrated by the recent finding that binding of Oct4 and CTCF to a composite element of the Xist gene is required for the regulation of X chromosome inactivation [25] (Fig. 7). Nevertheless, there is no overall correlation of combined binding of CTCF and Oct4 in the genome [26]. Likewise, we do not observe an enrichment of the Oct4 motif in the vicinity of CTCF binding regions as compared to random genomic regions (see Fig. 1A).

For the functional characterization of the TR/CTCF composite elements we used the episomal system. Episomal constructs are able to mimic endogenous chromatin and can be used for insulator analysis [43]–[46]. We found that for the episomal vectors that we constructed, the analysis of enhancer blocking is also possible. Addition of T3 relieves enhancer blocking, an important control that demonstrates that the episomal system can be used to study T3 regulated enhancer blocking. Thereby we identified a new composite element at the *ESRRA* gene, which indeed mediates T3 regulation of enhancer blocking. Furthermore, CTCF and inhibition of histone deacetylation showed similar effects in this system as has been demonstrated for endogenous genes. Both the enhancer blocking activity of CTCF as well as the transcriptional repression of rDNA genes by CTCF require poly(ADP)ribosylation [36], [47]. Tumor suppressor silencing has been recently shown to be caused by a deficiency in poly(ADP)ribosylation [37]. In line with these results we could demonstrate that episomal enhancer blocking mediated by a composite binding site is sensitive to a poly(ADP)ribosylation inhibitor as well. Furthermore, changes in histone acetylation in the context of enhancer blocking or insulation have been shown. For CTCF binding sites, an increase in acetylation of histone H3 as well as of H4 has been reported [48], [49]. In contrast, CTCF binding to histone deacetylase activity has been found as well [35], and functional episomal tests of the β globin insulator demonstrated a general histone deacetylation in the region extending from the enhancer to the gene [43].

Why is the ESRRA gene down-regulated after T3 treatment, whereas the assay shown above indicates a loss of enhancer blocking by T3? We can only speculate that from the different promoters of the gene one may be induced, which may cause other promoters to be turned of.

This assay revealed for the first time that in the case of two composite elements a functional synergy between CTCF and the unliganded TR confers enhancer blocking. This shows that, similar to enhancer elements which are comprised of functional modules (binding sites for enhancer factors), enhancer blockers are generated from functional and synergizing modules as well (Fig. 7). Again, in analogy to enhancer elements, functional modules can be multimers of identical factors (CTCF) as in the case of the H19 locus [50], [51] or in the X inactivation locus of the active mammalian X chromosome [15], or different factors such as combinations of CTCF with Oct4 [25], Kaiso [52], Yy1 [53] or TR, as shown here. In these cases (Fig. 7), binding of both factors involved is required to mediate the biological function.

## Materials and Methods

### Motif scanning

Half site models for nuclear receptor were downloaded from TRANSFAC database. Next we built PSSMs with the appropriate spacing (ER6, DR4, DR0, IR4). The PSSM for CTCF was derived from published genomewide CTCF binding data [20] by scanning the top 1000 binding regions with the MEME tool [54]. The PSSM for the combined Oct4/Sox2 sites was taken from Li et al. [55]. PSSMs were used to scan 13720 described CTCF binding regions from −500 bp to +500 bp relative to individual peak centers using the Patser tool of the RSAT suite [22]. In case of the NR half site models we compared the performance of our prediction method with the NHR-Scan and NUBIscan tools [23], [24] on random 1 Mb regions and found identical motif prediction in >80% of the cases. Random control datasets were generated with custom BioPerl scripts from the repeat masked human genome downloaded from UCSC (http://hgdownload.cse.ucsc.edu/goldenPath/hg18/bigZips/). 2000 repetitions of such control experiments were conducted. To control for biases in the sequence composition of the CTCF binding regions we shuffled the base positions of the PSSMs and used these to scan the 13720 CTCF binding regions. In case of the NR half sites models we tested all possible permutations (720) using the same permutation in both half sites. For the CTCF and Oct4/Sox2 PSSMs we tested the same number of random permutations. Empirical p-values were calculated as the fraction of simulations that produced a number of mapped features as extreme as observed in the real data [56].

### Plasmid construction

A second MCS (PstI, EcoRI, BglII, EcoRV, Spe) was inserted into the ClaI site of the pGL3-control vector (Promega). pR-E was generated by digesting the pGL3-MCSII vector with BamHI fill in and NheI and cloned in NotI fill in and NheI cut pREP4-ss. pREP4-ss is a pREP4 vector (Invitrogen), where the RSV LTR promoter was removed. The F1F2 element of the chicken lysozyme gene was amplified with primer pairs containing SpeI and BglII restriction sites. The PCR product was cloned into the BglII and SpeI sites of the MCSII of the pGL3-MCSII vector generating pGL3F1F2. pRF1F2E was generated by digesting the pGL3F1F2 vector with BamHI and XhoI, and was cloned blunt end into pREP4-ss NotI blunt ended. The F1F2 element was multimerized by ligating PCR products amplified from the pGL3F1F2 vector with primers containing SpeI and XbaI restriction sites. The 5xF1F2 was subcloned blunt into pBK-CMV (pBK-CMV5xF1F2), further cloned as a XbaI and BamH1 fragment into pGL3-MCSII digested with SpeI and BglII (pGL3-5xF1F2) and again cloned as a BamH1/XhoI fragment into pREP4-ss NotI blunt to generate pR5xF1F2E. pRF1mutF2E was generated as follows: Primer containing the F1mut binding site were annealed and directly cloned into the XbaI, HindIII site of pBK-CMV (Stratagene). Primer containing the F2 element were annealed and directly cloned into the HindIII, BamHI site of pBK-CMV F1mut. pGL3F1mutF2 was generated by digested pBK-CMVF1mutF2 with XbaI and SpeI and cloning the fragment into the SpeI site of pGL3-MCSII. pRF1mutF2E was generated by digesting pGL3-F1mutF2 with SalI fill in and NheI and cloned into pREP4-ss cut with NotI fill in and NheI. pRF1F2mutE was cloned in the same manner with F1 and F2mut oligos.

pRCOMTE was generated by PCR amplification of the genomic locus with corresponding primer pairs containing SpeI and XbaI restriction sites and subcloned into pBSK (Stratagene) generating pBSK-COMT. To multimerize binding sites pBSK-COMT was cut with SpeI and NotI and ligated into pBSK-COMT cut with XbaI and NotI. pBSK-COMT was digested with with XbaI and SpeI and cloned into pGL3-MCSII digested with SpeI to generate pGL3-COMT and again cloned as a BamH1 fill in NheI fragment into pREP4-ss digested with NotI fill in and NheI. All other newly identified CTCF and TR binding sites were cloned in the same manner. To generate enhancer-less episomes the corresponding pGL3-vector was cut with EcoRV and NheI and cloned into pREP4-ss digested with NotI fill in and NheI.

### Cell culture and transfection

The clonal cell line Neuro-2a stably transfected with the β1 isoform of the human thyroid hormone receptor [57] (N2a-β cells), HepG2 (ATCC HB-8065™) and HEK293T (ATCC CRL-11268™) were grown in Dulbecco's modified Eagle's medium supplemented with 10% (v/v) charcoal stripped hormone-depleted serum and 1% PenStrep.

N2aβ and 293T cells were transfected using the calcium phosphate method essentially as described [58]. In detail, cells were transfected with 1 µg reporter plasmid per 6-well dish or as indicated. 24 h after transfection T3 (10^−6^ M) was added to the medium for 48 h before harvesting cells.

For TSA experiments, TSA (BIOMOL) was added 8 h after transfection at a concentration of 10 ng/ml. Cells were harvested after 40 h of TSA treatment.

3-ABA (Sigma) was added 28 h after transfection at a final concentration of 8 mM. and cells were collected 14 h later.

### Electrophoretic mobility shift assay

Radiolabeled DNA probes (Table S1) were generated by phosphorylation with gamma ^32^P ATP and subsequently annealed. The probes were incubated with 0.5–2 µg of purified GST, GST-CTCF-ZF and human GST-TR. Recombinant proteins were prepared as described previously [59]. The binding reaction was performed in PBS ([pH 7.4], supplemented with 5 mM MgCl_2_, 1 mM ZnCl_2_, 1 mM DTT, 0.1% NP-40 and 10% glycerol) for 20 min at room temperature in the presence of 200 ng/µl pdIdC and 25–100 ng/µl salmon sperm DNA. Protein-DNA complexes were analyzed on nondenaturing polyacrylamide gels (5% acrylamide [w/v]) in TAE-buffer. Electrophoresis was performed at 4°C with a field strength of 12 V/cm for 3–4 h.

### Chromatin immunoprecipitation

ChIP assays were performed as previously described [60] with following modifications: 1×10^7^ HeLa-cells (ATCC CCL-2™) in 1 ml SDS lysis buffer were sonicated 7 times on ice with a Branson 250 sonifier on setting 1, constant for 10 secs to an average length of approximately 300–800 bp. Sonicated chromatin from 2×10^6^ cells was diluted 10-fold in ChIP dilution buffer and precleared with 30 µl of salmon sperm DNA/protein A agarose solution (Santa Cruz #16-157) and 5 µg preimmune serum for 2 hrs before overnight incubation at 4°C with antibody or controls.

For immunoprecipitation we used antibodies specific for CTCF [61] and preimmune serum. Gene specific PCR mixtures contained 1 µl of DNA, 0.5 µM of each primer (Table S1); 1.5 mM MgCl_2_, 0.2 mM dNTPs, and 1.25 U of Taq DNA polymerase (Panscript) in a total volume of 35 µl. Following 32 to 37 cycles of amplification, PCR products were run on a 3% agarose gel and analyzed by ethidium bromide staining. Annealing temperatures and cycling conditions were determined empirically for each primer set.

### GST-pulldown

GST and GST fusion proteins were expressed in *Escherichia coli* BL21. Bacteria were induced with 0.5 mM isopropyl-d-thiogalactopyranoside for 5 h at RT. Recombinant proteins were purified with glutathione–sepharose beads (Amersham Pharmacia Biotech AB) and analyzed on SDS–PAGE to normalize protein amounts (Fig. S4). Equivalent amounts of GST fusion proteins were incubated with [35S]methionine-labeled proteins, produced by the T7\T3 TNT-coupled transcription/translation system (Promega) in 200 µl of binding buffer. After 2 h incubation at 4°C the beads were washed 3 times with 1 ml of binding buffer without BSA. The bound proteins were eluted with SDS sample buffer, fractionated on SDS–PAGE visualized by fluorography.

## Supporting Information

Figure S1 ChIP-assay demonstrates in vivo binding of CTCF. ChIP was performed using chromatin from HeLa cells and immunoprecipitated using antibodies against CTCF. Specific primers (see Table S1) for the CTCF target sites (CTS) were used in the PCR-reaction. Negative controls: nonspecific antibody (IGG) and a nonbinding sequence (ESRRα-control).
(0.26 MB TIF)

Figure S2 Specificity of in vitro binding of TRβ to predicted target sites. EMSA experiments were performed using E.coli expressed GST and GST-TR with the indicated radioactively labeled probe. For competition experiments a 0.5 or 50-fold molar excess of non-labeled TR binding site (F2) or unspecific probe (N) were used. Arrow marks the TR specific shift.
(0.24 MB TIF)

Figure S3 Tetracycline inducible shRNA-mediated knock-down of CTCF. Protein levels were measured by western blotting using an anti-CTCF antibody or GAPDH as control. 293T cells of each transfected sample (see Fig. 5B) were collected and analyzed by 7.5% SDS PAGE. The CTCF knockdown results in a reduction of about 50%.
(0.25 MB TIF)

Figure S4 Coomassie stained gel shows expression levels of GST-fusion proteins. Asterisks mark the corresponding GST-fusion proteins.
(0.75 MB TIF)

Table S1 List of oligonucleotides used in different applications.
(0.12 MB DOC)
