# Supplementary figures and images for: Modular Insulators: Genome Wide Search for Composite CTCF/Thyroid Hormone Receptor Binding Sites
Source: PLoS One. 2010 Apr 9;5(4):e10119. doi: 10.1371/journal.pone.0010119 (PMC2852416; doi:10.1371/journal.pone.0010119)

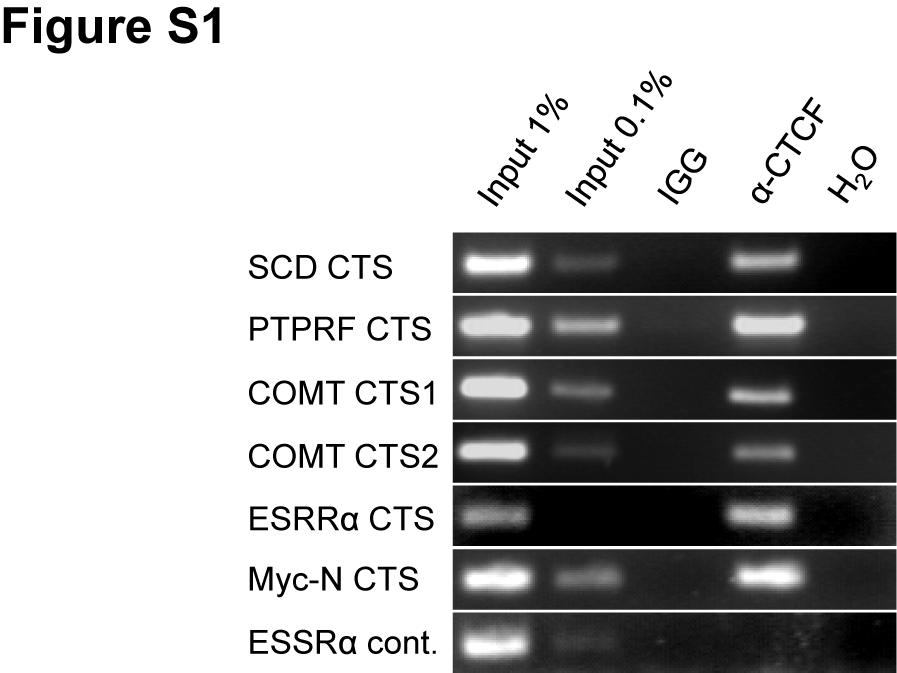

Supplement: Figure S1 — ChIP-assay demonstrates in vivo binding of CTCF. ChIP was performed using chromatin from HeLa cells and immunoprecipitated using antibodies against CTCF. Specific primers (see Table S1) for the CTCF target sites (CTS) were used in the PCR-reaction. Negative controls: nonspecific antibody (IGG) and a nonbinding sequence (ESRRα-control). (0.26 MB TIF) [file pone.0010119.s001.tif]

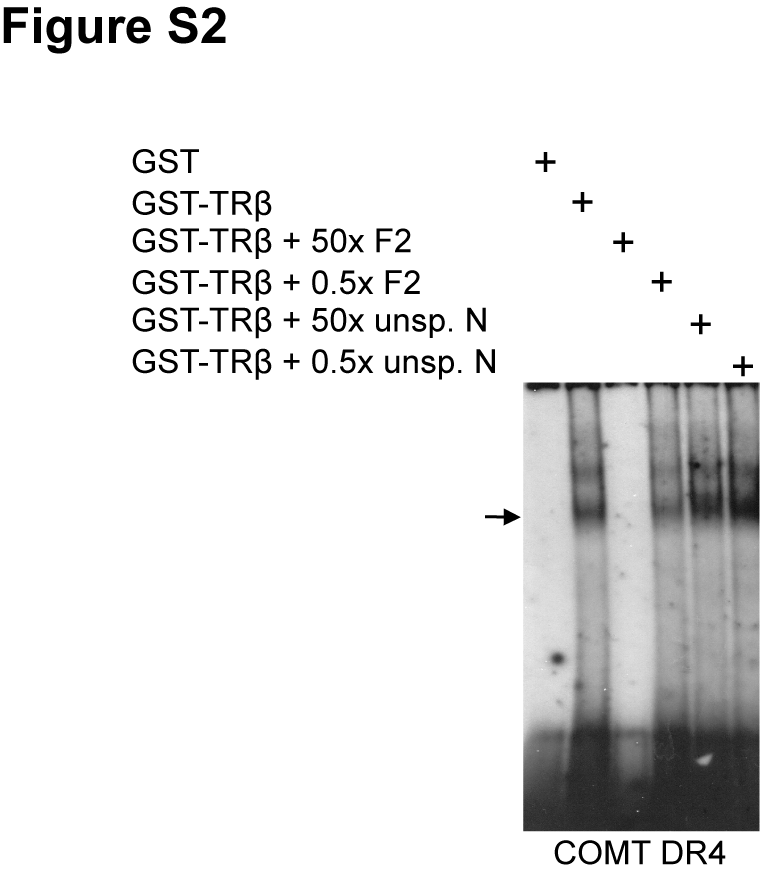

Supplement: Figure S2 — Specificity of in vitro binding of TRβ to predicted target sites. EMSA experiments were performed using E.coli expressed GST and GST-TR with the indicated radioactively labeled probe. For competition experiments a 0.5 or 50-fold molar excess of non-labeled TR binding site (F2) or unspecific probe (N) were used. Arrow marks the TR specific shift. (0.24 MB TIF) [file pone.0010119.s002.tif]

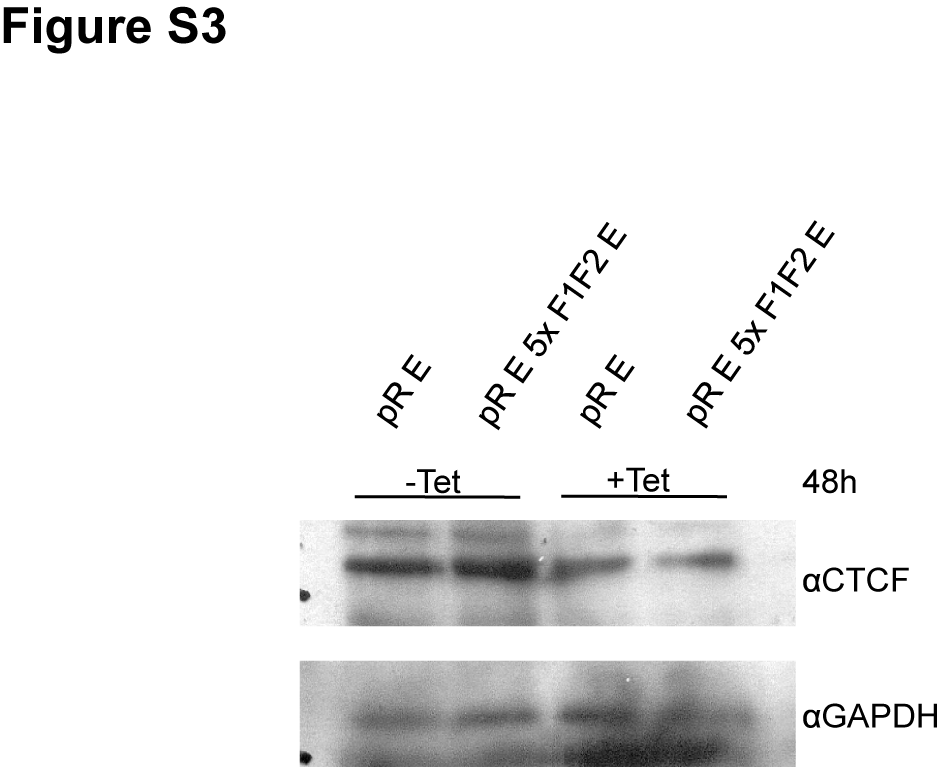

Supplement: Figure S3 — Tetracycline inducible shRNA-mediated knock-down of CTCF. Protein levels were measured by western blotting using an anti-CTCF antibody or GAPDH as control. 293T cells of each transfected sample (see Fig. 5B) were collected and analyzed by 7.5% SDS PAGE. The CTCF knockdown results in a reduction of about 50%. (0.25 MB TIF) [file pone.0010119.s003.tif]

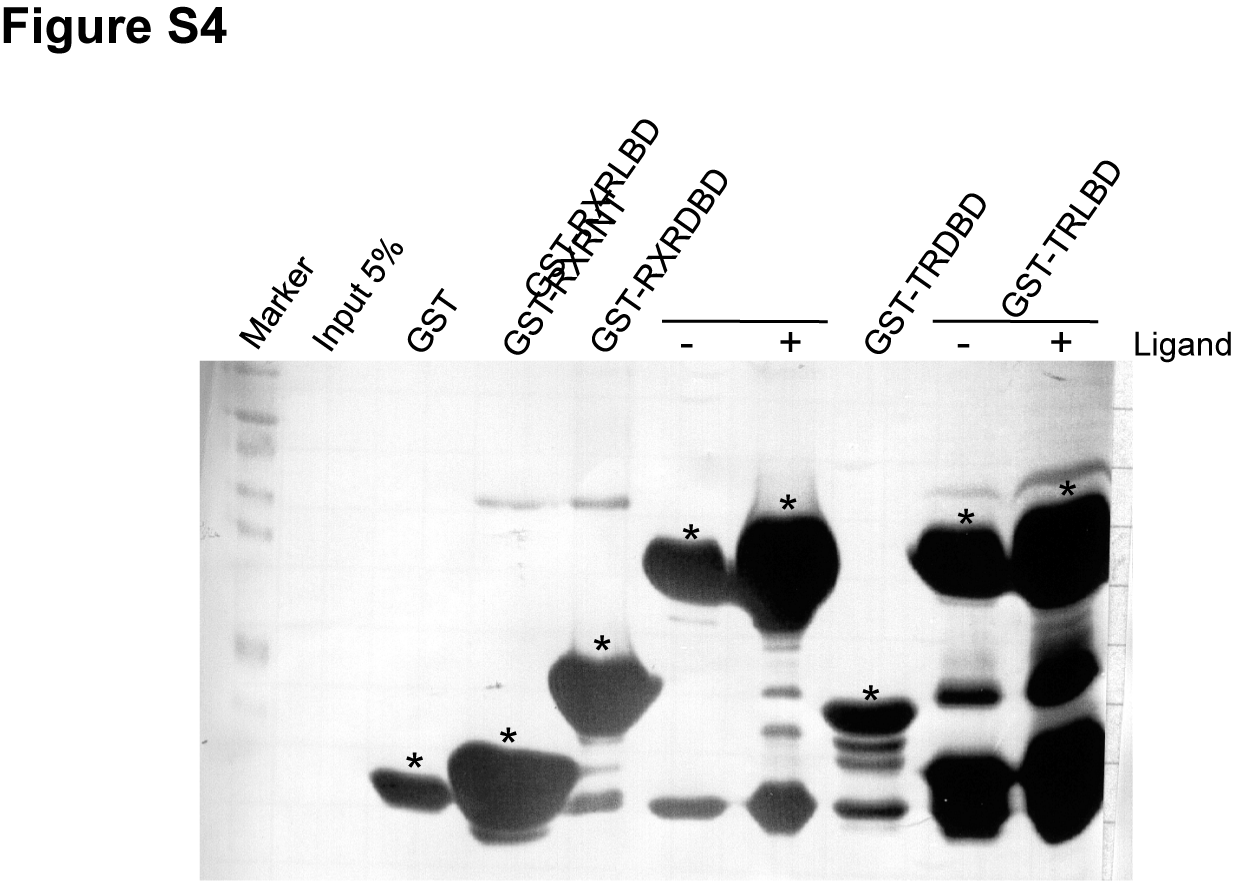

Supplement: Figure S4 — Coomassie stained gel shows expression levels of GST-fusion proteins. Asterisks mark the corresponding GST-fusion proteins. (0.75 MB TIF) [file pone.0010119.s004.tif]
