# Supplementary material for: Modular Insulators: Genome Wide Search for Composite CTCF/Thyroid Hormone Receptor Binding Sites
Source: PLoS One. 2010 Apr 9;5(4):e10119. doi: 10.1371/journal.pone.0010119 (PMC2852416; doi:10.1371/journal.pone.0010119)
Supplement: Table S1 — List of oligonucleotides used in different applications. (0.12 MB DOC) [file pone.0010119.s005.doc]

**Table S1:** Primer sequences

| **Application** | **Primer name** | **Sequence** |
| --- | --- | --- |
| **ChIP** | | |
|  | SCD CTS s | cagaacaaattcttcactcctacct |
|  | SCD CTS as | tgtggagaaaagtggtctctagatt |
|  | PTPRF CTS s | ggtgaaaaaccactcactggaa |
|  | PTPRF CTS as | ttcagggatcacgtggtaatg |
|  | COMT CTS1 s | aacagcctgagtccgtgtctg |
|  | COMT CTS1 as | ctttgcttggagtgccacc |
|  | COMT CTS2 s | acagctgccaacagcagagg |
|  | COMT CTS2 as | gccagaggcacacacctgct |
|  | ESRRα CTS s | atctgcggttcctcgaatca |
|  | ESRRα CTS as | atgtatcgctgcagctcctt |
|  | MycN CTS s | tgttttaaggaaccgcctgt |
|  | MycN CTS as | cgttcaggtttgcgaaagta |
|  | ESSRα control s | ttatgatttgcgctggttga |
|  | ESSRα control as | gcacacagcaagtgctcaat |
| **EMSA** | | |
|  | F1 CTS s | CTAGATGAAGAAATTGAGACCTCTACTGGATAGCTATGGTATTTACGTGTCTA |
|  | F1 CTS as | AGCTTAGACACGTAAATACCATAGCTATCCAGTAGAGGTCTCAATTTC TTCAT |
|  | F2 TRE s | AGCTTTTAGTTACTTATTGACCCCAGCTGAGGTCAG |
|  | F2 TRE as | GATCCTGACCTCAGCTGGGGTCAATAAGTAACTAAA |
|  | F1_mut_ CTS s | CTAGATGAAGAAATTGAGATTTCTACTGGATAGCTATGGTATTTACGTGTCTA |
|  | F1_mut_ CTS as | AGCTTAGACACGTAAATACCATAGCTATCGAGTAGAAATCTCAATTTC TTCAT |
|  | F2_mut_ TRE s | AGCTTTTAGTTACTTATTGGGTCCAGCTGATCCCAG |
|  | F2_mut_ TRE as | GATCCTGGGATCAGCTGGACCCAATAAGTAACTAAA |
|  | COMT CTS1 s | cctgctctgtctacccgagggcaccagagggcacgagaaggctggctccctg |
|  | COMT CTS1 as | cagggagccagccttctcgtgccctctggtgccctcgggtagacagagcagg |
|  | COMT CTS2 s | tgaaccttgcccctctgcaaacacaagggggcgatggtggcactccaagcaa |
|  | COMT CTS2 as | ttgcttggagtgccaccatcgcccccttgtgtttgcagaggggcaaggttca |
|  | SCD CTS s | ggtgctttacctaccagggccactgggtggcagcaggaccctggccccat |
|  | SCD CTS as | atggggccagggtcctgctgccacccagtggccctggtaggtaaagcacc |
|  | PTPRF CTS s | cagtcccatcccctcctcatacgccagggggcgcacacgcactgtttgtgaa |
|  | PTPRF CTS as | ttcacaaacagtgcgtgtgcgccccctggcgtatgaggaggggatgggactg |
|  | NR2F1 CTS s | tttaggcagcgaggactcttacactagggggcagacagatactgtacttgct |
|  | NR2F1 CTS as | agcaagtacagtatctgtctgccccctagtgtaagagtcctcgctgcctaaa |
|  | ESRRα CTS s | ggttcctcgaatcacacatgcggcgccccctggtggggagacctttcggt |
|  | ESRRα CTS as | accgaaaggtctccccaccagggggcgccgcatgtgtgattcgaggaacc |
|  | PER1 CTS s | cccattaccaactcctagtgcccccctgtggccactttgagaatcacact |
|  | PER1 CTS as | agtgtgattctcaaagtggccacaggggggcactaggagttggtaatggg |
|  | LRP1 CTS s | aacagccataaaacgccgcccagaagggggcagtgaccaaaagcacgttc |
|  | LRP1 CTS as | gaacgtgcttttggtcactgcccccttctgggcggcgttttatggctgtt |
|  | BCL3 CTS s | gaaggcgtgcccatggcaaccgctaggtggtgcccgtacaccaccagcca |
|  | BCL3 CTS as | tggctggtggtgtacgggcaccacctagcggttgccatgggcacgccttc |
|  | LMNA CTS s | GGTCACTGGCCTCCCTCCCGCCCCCTTCAGGACATTCTACTATCTTCTTA |
|  | LMNA CTS as | TAAGAAGATAGTAGAATGTCCTGAAGGGGGCGGGAGGGAGGCCAGTGACC |
|  | PCK1 CTS s | aaccatgctcagccacagtggcctctgcaggttgaggagggattcgctgt |
|  | PCK1 CTS as | acagcgaatccctcctcaacctgcagaggccactgtggctgagcatggtt |
|  | COMT DR4 s | acccgagggcaccagagggcacgaga |
|  | COMT DR4 as | tctcgtgccctctggtgccctcgggt |
|  | COMT IR4 s | cgtccagagcatgggtgaccagcacg |
|  | COMT IR4 as | cgtgctggtcacccatgctctggacg |
|  | SCD DR4 s | actcctgacctcaggtgatccaccca |
|  | SCD DR4 as | tgggtggatcacctgaggtcaggagt |
|  | PTPRF DR5 s | ttgtgaattcaagttcaggacaaaaac |
|  | PTPRF DR5 as | gtttttgtcctgaacttgaattcacaa |
|  | PTPRF DR4 s | actcctgacctcgggtgatctgcccg |
|  | PTPRF DR4 as | cgggcagatcacccgaggtcaggagt |
|  | NR2F1 IR4 s | tactgagttcagagttaaactcggca |
|  | NR2F1 IR4 as | tgccgagtttaactctgaactcagta |
|  | NR2F1 ER6 s | aattctgtgctctgtccagaacattcaa |
|  | NR2F1 ER6 as | ttgaatgttctggacagagcacagaatt |
|  | NR2F1 ER6 DR0 s | agatactgtacttgctctaatctt |
|  | NR2F1 ER6 DR0 as | aagattagagcaagtacagtatct |
|  | ESRRα DR0 s | ccgcagtgaccttgagctttctcc |
|  | ESRRα DR0 as | ggagaaagctcaaggtcactgcgg |
|  | ESRRα DR4 s | cgcgatgtccttttgtgtcctacaag |
|  | ESRRα DR4 as | cttgtaggacacaaaaggacatcgcg |
|  | PER1 DR4 5’ s | actcctgacctcaaatgatctgcctg |
|  | PER1 DR4 5’ as | caggcagatcatttgaggtcaggagt |
|  | PER1 DR4 3’ s | acaagagatcaattgtggccaacccc |
|  | PER1 DR4 3’ as | ggggttggccacaattgatctcttgt |
|  | LRP1 ER 6 s | ggcagtgaccaaaagcacgttcactggc |
|  | LRP1 ER6 as | gccagtgaacgtgcttttggtcactgcc |
|  | BCL3 DR4 s | caggaggatcacctgaggtcagaaat |
|  | BCL3 DR4 as | atttctgacctcaggtgatcctcctg |
|  | LMNA ER6 s | ctaaatgtccctcgttttggtcactggc |
|  | LMNA ER6 as | gccagtgaccaaaacgagggacatttag |
|  | PCK1 IR4 s | gcccaagcacaaatgtgaccctcggt |
|  | PCK1 IR4 as | accgagggtcacatttgtgcttgggc |
|  | PCK1 DR0 s | caaatgtgctcttgagcctatttt |
|  | PCK1 DR0 as | aaaataggctcaagagcacatttg |
|  | GHR DR5 s | GTATTTGAGCCACAGCTGAGCTTCTGA |
|  | GHR DR5 as | TCAGAAGCTCAGCTGTGGCTCAAATAC |
|  | GHR DR4 s | CGGCGTGACCCCTGGTGAACGGTGGC |
|  | GHR DR4 as | GCCACCGTTCACCAGGGGTCACGCCG |
| **Cloning** | | |
|  | LMNA Spe | tttactagtggcttcttgtgaagggaatcc |
|  | LMNA Xba | ttttctagagcaaaagaagtagtgcctggag |
|  | PCK1 Spe s | tttactagtctcagttaaaatgccgcattg |
|  | PCK1 Spe as | tttactagtcctttcttcatctgggtatgacatt |
|  | ESRRα Spe | tttactagtatctgcggttcctcgaatca |
|  | ESRRα Xba | ttttctagaggtgggtgaagccgacttaaa |
|  | LRP1 Spe | tttactagtgcagacacctagaaagtcagacact |
|  | LRP1 Xba | ttttctagagatactttgcagaaggcaggc |
|  | COMT Spe | tttactagttgagtccgtgtctgcttctgt |
|  | COMT Xba | ttttctagactttgcttggagtgccac |
|  | NR2F1 Spe | tttactagtctgacttaagaagcaattctgtgc |
|  | NR2F1 Xba | ttttctagaggtcctctgaaaaatttacagtgct- |
|  | BCL3 Spe | tttactagtaagctccggatggaaggcgt |
|  | BCL3 Xba | ttttctagaccgtgttggccaggtttgt |
|  | PTPRF Spe | tttactagtggtgaaaaaccactcactggaa |
|  | PTPRF Xba | ttttctagattcagggatcacgtggtaatg |
|  |  |  |
|  |  |  |
|  |  |  |
|  |  |  |
